# Dispersion of nonresonant third-order nonlinearities in Silicon Carbide

**DOI:** 10.1038/srep40924

**Published:** 2017-01-18

**Authors:** Francesco De Leonardis, Richard A. Soref, Vittorio M. N. Passaro

**Affiliations:** 1 Dipartimento di Ingegneria Elettrica e dell’Informazione, Politecnico di Bari Via Edoardo Orabona n. 4, 70125 Bari, Italy; 2 Department of Engineering, The University of Massachusetts, Boston, Massachusetts, 02125 USA

## Abstract

In this paper we present a physical discussion of the indirect two-photon absorption (TPA) occuring in silicon carbide with either cubic or wurtzite structure. Phonon-electron interaction is analyzed by finding the phonon features involved in the process as depending upon the crystal symmetry. Consistent physical assumptions about the phonon-electron scattering mechanisms are proposed in order to give a mathematical formulation to predict the wavelength dispersion of TPA and the Kerr nonlinear refractive index n_2_. The TPA spectrum is investigated including the effects of band nonparabolicity and the influence of the continuum exciton. Moreover, a parametric analysis is presented in order to fit the experimental measurements. Finally, we have estimated the n_2_ in a large wavelength range spanning the visible to the mid-IR region.

There are over two hundred chemically stable semiconducting polytypes of silicon carbide (SiC) that have a high bulk modulus and a generally wide band gap. Among the different polytypes, numerous hexagonal (H) and rhombohedral (R) structures of SiC have been identified in addition to the common cubic form (C). In this context, the most common and technologically advanced SiC polytypes are 4H-SiC and 6H-SiC with wurtzite structures, and 3C-SiC with a zinc-blende crystal structure. These three polytypes form the focus of this paper. Their versatility is seen in the context of their semiconductor processing protocols. Indeed their processing is compatible with industrial standards– leading to the realization of SiC nanostructures such as nanoparticles, quantum dots, nanowires and nanopillars. In addition, the ability to grow epitaxially high-quality SiC crystal on different substrates, most notably on silicon^1^, provides advantages that facilitate the fabrication of nanophotonic cavities^2^,^3^. For these reasons and because of its unique physical properties and mechanical/chemical stability, SiC is now considered to be a promising platform for electronic and photonic applications. Indeed, the wide bandgap (from 2.4 to 3.2 eV, depending on the polytype), the high thermal conductivity, the ability to sustain high electric fields before breakdown and the highest maximum current density make SiC material ideal for realizing microelectronic devices operating under high-power conditions. In addition, the strong bonding between Si and C atoms in SiC makes this material very resistant to high temperature and radiation damage. As a result, the SiC platform offers large potential for realizing devices in radiation-hard applications^4^,^5^,^6^,^7^,^8^.

Another interesting field of applications for the SiC platform concerns the optical limiting devices for high power laser radiation. Of special interest are those limiters used in optical communication areas such as optical switching or laser beam control. Also important aresensitive eye protectors/detectors that work under extremely aggressive conditions such as high and low temperatures, high levels of light and radiation power, and chemical atmosphere. With their highly nonlinear optical properties, SiC materials are ideal for realizing the kinds of devices just described. In this context, SiC is a promising material because of its high optical and mechanical strength, thermal stability, chemical inactivity and large optical nonlinearities just mentioned. The second order nonlinear susceptibility χ^(2)^ has been observed in some nanostructured 6H-SiC layers^9^,^10^. Additionally, a high third-order optical nonlinear susceptibility χ^(3)^ ∼ 105 esu has been obtained in refs 11 and 12 by using thin films of 21R and 27R polytypes. Recently, optical limiting effects in β-SiC(3C) nanostructured thin films have been demonstrated by means of the Z-scan-like technique^13^. In that work, the authors have estimated a two photon absorption coefficient (*β*^*TPA*^) of 0.5462 and 0.4371 cm/kW at the laser wavelength of 532 and 1064 nm, respectively. Moreover, nonlinear refractive index (*n*_2_) values of 2.7 × 10^−5^ and 0.919 × 10^−5^ esu have been recorded at the same wavelengths. However, the authors point out that the nanostructured films exhibited a nonlinear effect four orders of magnitude higher than that in bulk 3C-SiC.

Recently, the novel demonstration of a passively mode-locked erbium-doped fiber laser (EDFL) based on a nonstoichiometric silicon carbide (Si_x_C_1−x_) saturable absorber has been reported^14^. In particular, the authors have shown that when the C/Si composition ratio is increased to 1.83, the dominant absorption of the Si_x_C_1−x_ film changes from two-photon absorption (TPA) to nonlinear saturable absorption, and the corresponding TPA value falls to ~3.9 × 10^−6^ cm/W. On the contrary, if a Si-rich Si_x_C_1−x_ is adopted, the film cannot mode lock the EDFL because it induces high intracavity loss through the TPA effect.

Looking at major applications of the SiC platform, it seems plausible to suppose that this platform can provide a boost to Si-based Group IV photonics. The past decade has seen tremendous developments in Group-IV photonics where silicon (Si) and germanium (Ge) and SiGeSn group-IV semiconductors have been widely used for advanced optoelectronic linear and nonlinear photonic applications. Heretofore, none of Group IV platforms based on CMOS-compatible exhibits simultaneously a large bandgap, a bulk second-order susceptibility χ^(2)^, a high third-order χ^(3)^ susceptibility and a refractive index above 2.0. However, this desired combination of characteristics could be realized in the SiC platform, especially devices enabled by “SiC-on-insulator” wafers (SiC/SiO_2_/Si) that would offer linear and nonlinear photonic devices at visible wavelengths. Moreover, unlike silicon and germanium, SiC does not suffer from any two photon absorption effects in the near (NIR) and mid-infrared (Mid-IR) regions due to its large bandgap. That is why SiC is a promising nonlinear platform in that IR range. In this infrared context, the first demonstration of the self-phase modulation (SPM) effect in a 4H-SiC channel waveguide has been reported in ref. 15, where the Kerr nonlinear refractive index *n*_2_ = 8.6 × 10^−15^ cm^2^/W has been estimated for a pump beam centered at 2360 nm.

The limited experimental data^15^,^16^,^17^,^18^ available for the SiC wurtzite structure indicate that the silicon carbide crystals can exhibit a strong third-order nonlinear susceptibility, χ^(3)^, comparable to that in Si, depending upon the operation wavelength. Overall, the nonlinear optical (NLO) response of 3C, 4H and 6H SiC structures could open up a wide range of applications such as four-wave mixing, wavelength conversion, third harmonic generation, infrared parametric amplification, frequency comb generation, continuum generation, and self-phase modulation, covering the optical spectrum from the visible through the mid infrared.

However, third-order NLO photonics based on the SiC platform is still an open issue. Indeed, to develop the full potential of SiC as a new technological platform for on-chip integrated nonlinear optical devices, it is crucial to have knowledge of the wavelength dispersion of the relevant third-order NLO coefficients (i.e. *β*^*TPA*^ and *n*_2_).

To the best of our knowledge, experimental knowledge of these coefficients is missing in the literature for a large wavelength range, thus preventing the extraction of a wavelength-dispersion law. To remedy this deficiency and to determine the desired dispersion curves, we have addressed here the relevant physics problems using a non-trivial physical model in order to estimate these nonlinear coefficients.

The paper is organized as follows. A new theoretical formulation is reported in Section 2 in order to estimate the TPA coefficient and the Kerr effect. In particular, the aim of this section is to describe the main physical effects that can influence the TPA process in SiC material. Then, the selection rules based upon group theory are introduced for zincblende and wurtzite silicon carbide crystals in order to find the allowed transitions and the electron-phonon interaction involved in the TPA process. Theoretical assumptions are validated in Section 3, where our numerical estimations are compared with the experimental data of the 4H-SiC semiconductor. Finally, Section 4 summarizes the conclusions.

## Theory

Silicon carbide has been the subject of many theoretical studies. In this context, a variety of structural, electronic and optical properties in SiC have been examined theoretically by many research groups and the results have been well related to the experimental measurements. However, to the best of our knowledge, efforts have not yet been made in the literature to investigate the physical features of the TPA process and the wavelength dispersion of the TPA and Kerr coefficients. To solve that deficiency, we propose a physical discussion for cubic and hexagonal polytypes of SiC in order to theoretically predict the third-order nonlinearity in that spectral range where experimental data are not available. Moreover, as outlined in ref. 15, only 3C-SiC, 4H-SiC, and 6H-SiC are generally used for fabrication of photonic channel waveguides. Crystalline SiC waveguides, wurtzite as well as cubic, are ideal for realizing on-chip the third-order nonlinear applications discussed above.

Along with low loss, there is a requirement on indices for the nonlinear strip waveguides. To provide the needed refractive index contrast between the SiC waveguide core and the transparent lower cladding (substrate), the cladding should have an index less than ~2.0. In fact, there are several ways in which to construct these cubic or wurtzite SiC channels, specifically from starting wafers of SiC/SiO_2_/Si or SiC/Si_3_N_4_/Si or SiC/Al_2_O_3_/Si. A successful fabrication technique has proven to be the “smart cut” method, in which an H-implanted oxide-coated 4H SiC wafer is bonded to an oxidized Si wafer^15^. 3C SiC has also been grown epitaxially upon sapphire for waveguiding^19^. An acceptable propagation loss of 7 dB/cm has been measured for 4H SiC on silica upon silicon. From resonator Q, an implied loss of 12 dB/cm has been observed for 3C-SiC-on-insulator channels^18^. Small-area heteroepitaxy of 3C SiC on Si has been used to reduce defects^20^, and this wafer preparation for smart cut may yield a waveguide loss comparable to that found for wurtzite waveguides.

Due to the very large value of the direct bandgap, we guess that SiC materials do not suffer from any TPA effect induced by direct transitions as dominated by the allowed-forbidden (a-f) transitions (as will be demonstrated in the following section). Thus, based on our previous theoretical work^21^, we investigate the nonlinear absorption processes as induced “only” by indirect transitions involving the intermediate states with Γ symmetry in the conduction band as well as nonlinear absorption by phonon emission and absorption.

**Figure 1:**
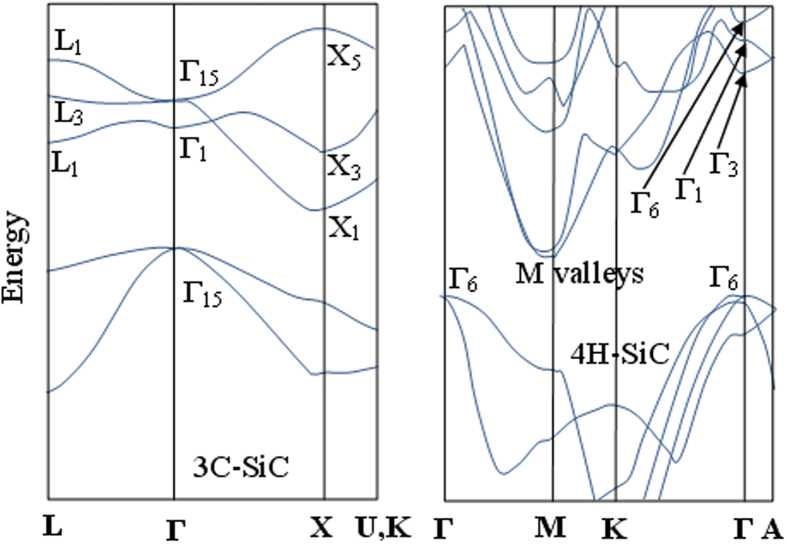
Schematic band diagram for 3C-SiC (left) and 4H-SiC (right).

In Fig. 1(a) and (b) the schematic band diagrams are shown for 3C-SiC and 4H-SiC bulk materials, respectively. The plots indicate the fundamental symmetry points useful in applying the group-theory selection rules.

Actually, an electron makes a transition from the doubly degenerate valence bands at **K** = 0, *v*_1_ (heavy hole) and *v*_2_ (light hole), to the minimum conduction band *c* (at X_1_ or M symmetry points for 3C-SiC and 4H-SiC, respectively) through the intermediate states, generally indicated as *n* and *m*^21^. Therefore, two photons at the frequencies *ω*_1_ and *ω*_2_ are absorbed to transit from the valence bands to the intermediate states, then a phonon of energy *E*_*ph*_ = *ħ*Ω is absorbed or emitted in order to complete the transition from one of the two intermediate states to the minimum conduction band.

According to the model detailed in our previous work^21^, under the hypothesis of parabolic bands the indirect TPA coefficient should be given by Eq. (1):


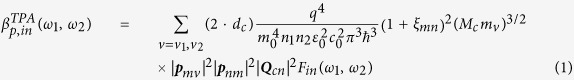


In particular, the term *F*_*in*_(*ω*_1_, *ω*_2_) is calculated as in Eq. (2):


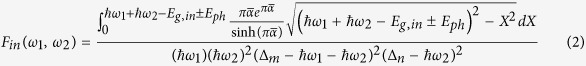


with 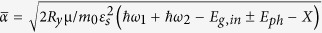, and the subscript (*in*) indicates the indirect transitions.

Generally speaking, the two-photon indirect absorption can also be influenced by the Coulomb interaction. In this sense, in Eq. (2), the term 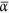 is related to the continuum exciton effect. However, if we assume the nonparabolicity of both valence and conduction bands, the TPA coefficient, 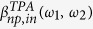, can be calculated as:


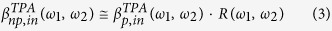


where the function *R*(*ω*_1_, *ω*_2_) is defined as in Eqs (4, (5), 6):


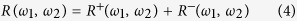



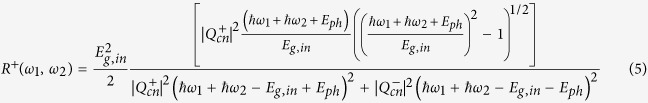



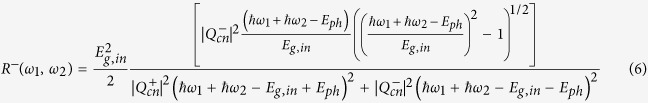


It is worth noting that Eq. (3) is rigorous in the absence of any continuum exciton influence. Moreover, the assumption of nonparabolicity applied only to the conduction band (see *R*(*ω*_1_, *ω*_2_) defined in ref. 21) gives very good results for several semiconductors (i.e., Si, Ge and GeSiSn alloy), but it represents too large of an approximation in the case of the silicon carbide materials. Indeed, our investigations indicate that the nonparabolicity of both valence and conduction bands effect constitutes a fundamental aspect of SiC structures, confirmed by a very good matching between theoretical predictions and the experimental data. For this reason, the *R*(*ω*_1_, *ω*_2_) function of Eqs (4), (5), (6) represents a generalization of that presented in ref. 21 by imposing the nonparabolicity effect on both conduction and valence bands.

In Eq. (1), *E*_*g,in*_, *m*_*v*_, *M*_*c*_ indicate the indirect bandgap energy, the hole (heavy or light) effective mass, and the electron effective mass, respectively. In particular, since the absorption process involves the indirect conduction valleys, the electron effective mass may be approximated by 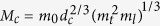, where *m*_*t*_, *m*_*l*_, and *d*_*c*_ represent the transverse mass, the longitudinal mass, and the number of equivalent conduction band minima, respectively^22^,^23^. Moreover, the coefficients *R*_*y*_ = 13.6 eV, Δ_*m*_, Δ_*n*_, and *ε*_*s*_ represent the Rydberg energy, the energy of the intermediate states *m* and *n*, and the semiconductor static dielectric constant, respectively. The terms 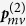 and 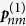 are the transition matrix element for the optical transitions 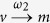, and 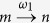, respectively. In addition, 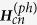 represents the electron-phonon interaction Hamiltonian satisfying the relationship 
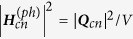
, with *V* the crystal volume and 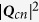 the matrix element for phonon scattering. Finally, the term *ξ*_*mn*_ indicates the permutation of the intermediate states. Indeed, the exchange in the order of the intermediate states can induce two different indirect transitions, (a) 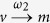, 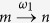, and 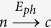, (b) 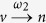, 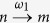, and 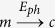. Now, *ξ*_*mn*_ can assume a value of 0 or 1 if one or both of the transitions are allowed by the selection rules. Thus, the physical features differ when moving from 3C-SiC to 4H-SiC. In this context, it is convenient to give the selection rules using the general method of group theory in order to take into account the crystal symmetry^24^. In case of 3C-SiC, the schematic diagram in Fig. 1(a) shows that the top of the valence state, the lowest conduction state, and the nearest intermediate states, have a symmetry as Γ_15_, X_1_ (indirect bandgap), Γ_1_, Γ_15_ (direct bandgap), and Γ_12_, respectively. Since 3C-SiC has a zinc-blende structure, the dipole operator acts with a symmetry Γ_15_. Thus, under the group theory picture, the following relationship holds:


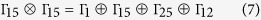


In the case of 3C-SiC, Eq. (7) indicates that, the most favorable two-photon indirect transitions involve two equivalent steps: 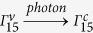, 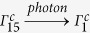, and 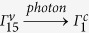, 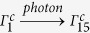. Thus, by referring to Eq. (1), the intermediate states indicated generically with *m* and *n* correspond to 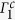 and 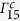, and the exchange among 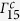, and 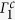 is allowed, resulting in *ξ*_*mn*_ = 1 (see Eq. (1)). Similarly, the group theory confirms Eqs (8) and (9):


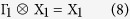



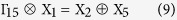


Thus, the transition 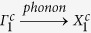 involves only the longitudinal acoustic (LA) phonons while in 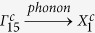 only transverse acoustic (TA) and transverse optical (TO) phonons are allowed. Consequently, we can conclude that all phonons are allowed in 3C-SiC except the longitudinal optical ones.

Similar to all of the known SiC polytypes, 4H-SiC is an indirect band-gap semiconductor. Using different standard notations, the space group is 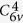 (Schoenflies notation) or *P*63*mc* (international notation), which is the character table at the Γ point given in ref. 25. According to a number of electronic band-structure calculations, the equivalent conduction-band minima are located at the point M of the Brillouin zone (see Fig. 1(b)). The maximum of the valence band is at the center of Γ. The symmetry of the conduction intermediate states, at the Γ point, will be Γ_6_ and Γ_1_ (in the BSW notation). The heavy hole and light hole bands will have symmetry Γ_6_. Furthermore, since the 4*H*-SiC semiconductor is an uniaxial crystal, the dipole operator can be decomposed in two components, parallel to the wurtzite unit cell optical axis (c-axis) with symmetry Γ_1_, and orthogonal to the c-axis with symmetry Γ_6_. We note that 4H-SiC and 6H-SiC are commercially available only as bulk crystalline wafers cut in on-axis or off-axis orientations. The on-axis orientation cut results in a wafer with c-axis perpendicular to its surface, with the ordinary, and extraordinary refractive index in the plane of the wafer, and perpendicular to the plane of the wafer, respectively. As evidenced clearly in ref. 15, this cut is ideal for photonic devices, since the TE polarization is aligned with the ordinary axes of the crystal and the TM polarization is aligned with the extraordinary axis, thereby preventing unwanted polarization rotation.

According to the group theory, the symmetry of the dipole operator acts on the valence bands as:


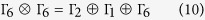



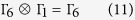


By inspecting Eqs (10) and (11), one recognizes that single optical transitions are allowed into 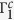 or 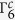 conduction bands, for light polarized parallel (TM polarization), or orthogonal (TE polarization) to the c-axis, respectively. However, in the frame of our TPA physical model, two allowed-allowed transitions involving two different intermediate states (see ref. 20 for detail) must be considered in order to apply Eq. (1). In this context, Eqs (10) and (11) indicate that the most favorable two-photon transitions for TE polarization are given by: 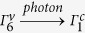, 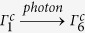. Thus, by referring to Eq. (1), the exchange among 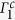, and 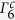 is forbidden, resulting in *ξ*_*mn*_ = 0. Conversely, when the light polarization is parallel to the c-axis (TM polarization), the optical transition 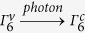 is allowed, while 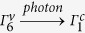 is forbidden. As a result, Eq. (1) is not directly applied. Thus, we guess that in the case of TM polarization the indirect TPA effect is characterized by intraband transitions in 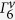, or 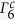, respectively. The following transitions: 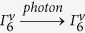 (intraband), 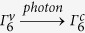, and 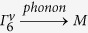 control the indirect TPA process with light polarization parallel to the c-axis. In this context, the 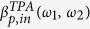 coefficient can be recalculated by assuming the photon transition matrix elements 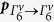, and 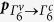 as being proportional to and independent from the wave-vector **K**, respectively (see the transition rate formula in ref. 21). However, since a reduced number of experimental data are relevant to the light polarization orthogonal to the c-axis, we adopt Eq. (1) to extract our numerical results.

Thus, in order to complete the physical analysis of indirect TPA effect in 4*H*-SiC crystals, it is important to investigate the nature of the phonons involved in the process. The phonons at the *M* point in the Brillouin zone can be classified by symmetry as *M*_1_, *M*_2_, *M*_3_, *M*_4_. The four irreducible representations are all one dimensional, and are listed as in Table 1 26.

According to the irreducible representation and the phonon dispersion calculation provided in ref. 26, the following observation can be made. There exists an energy gap in phonon dispersion, and the phonons of each symmetry are equally distributed above it (optical phonons) and below it (acoustic phonons). In particular, there are 8 phonons with *M*_1_ and *M*_4_ symmetry and 4 phonons with *M*_2_ and *M*_3_ symmetry, respectively^26^. Thus, the indirect TPA process for the TE polarization requires the electron-phonon interaction to induce the transition between the 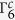 valley and the minimum conduction having symmetry *M*_4_ (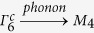). Thus, the group theory confirms Eq. (12):


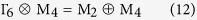


Consequently, we can conclude that in 4H-SiC all phonons with *M*_2_ and *M*_4_ symmetry are allowed in the indirect TPA process for TE polarization. Similar considerations hold for 6H-SiC material, in which the minima of conduction band occur in both *M* and *L* points.

Having attained the TPA coefficient, we now apply the Kramers-Kronig relationship in order to derive the Kerr nonlinear coefficient (*n*_2_) as:


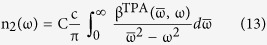


However, the functional expression of 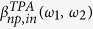, in Eqs (1), (2), (3), (4), (5), (6), does not lead us to derive a numerical solution for predicting the wavelength dispersion of *n*_2_. To get that solution, we adopt a number of approximations as follows. In particular, we neglect both the continuum exciton effect and photon energy with respect to the energies Δ_*m*_, and Δ_*n*_. Moreover, although the non-degenerate TPA coefficient is well defined by Eq. (1), the approximation^22^ of a degenerate TPA function with the substitution *ω* → (*ω*_1_ + *ω*_2_)/2 has been used. This approximation becomes progressively less accurate as the photon energy increases well above the half-bandgap, but it can give results reasonably close to the experimental values since it avoids mathematical and unphysical divergences at zero photon-energy. Under these assumptions, the integral of Eq. (13) admits of the solution given in Eq. (14), where the term *C*_*np*_ is a fitting coefficient depending on the explained approximations.


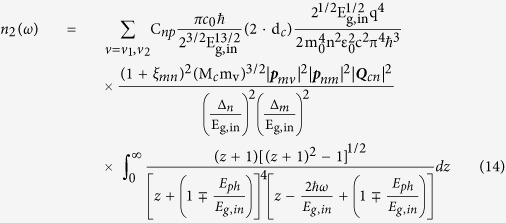


## Results

The goal of this section is to evaluate the theoretical wavelength dispersion for the third-order absorption occurring in 3C, 4H and 6H SiC polytypes. For that purpose, we apply the Kramers-Kronig relationship in order to estimate the nonlinear Kerr refractive index. The SiC physical parameters used in our simulation are listed in Table 2.

Furthermore, Sellmeier’s index equations for 3C-SiC^27^, 4H-SiC and 6H-SiC^28^ have been used in simulations to take into account the index dispersion of the material. Since Δ_*m*_ and Δ_*n*_ are not available in literature for the 6H-SiC polytype, we have assumed as references the values given for 4H-SiC. This assumption is in general not rigorous, however our parametric investigations indicate that small changes in Δ_*m*_, and Δ_*n*_ do not influence significantly the TPA dispersion, confirming the consistency of our approximation.

By referring to Eq. (1), knowledge of the 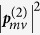, 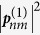, and |***Q***_*cn*_|^2^ parameters is required to obtain numerical values to be compared with experimental results. Generally speaking, these values can be derived from both electronic structure and electron-phonon scattering theoretical calculations. Additionally, experimental measurements could be used to better set the values of 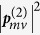, 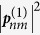, and |***Q***_*cn*_|^2^, in order to improve the model predictions of the third order nonlinearity in the wavelength range where experimental data do not exist. Although theoretical calculations of |***Q***_*cn*_|^2^ are difficult and unreliable, some preliminary numerical estimations can be derived by considering the acoustic phonon deformation potential and the intervalley phonon deformation potential scattering (see Eqs (15) and (16) in the Method section).

Due to the lack of a reasonable number of TPA coefficient measurements, we have carried out a set of parametric simulations in order to estimate, in a consistent way, the TPA wavelength dispersion. In this context, we adopt as a starting point the work proposed in ref. 17, in which the nonlinear absorption coefficient of 0.064 cm/GW has been measured at 780 nm for a semi-insulating 6H-SiC crystal.

**Figure 2:**
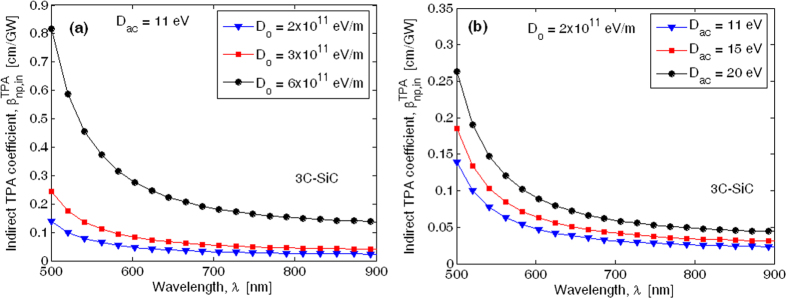
Spectra of degenerate two-photon absorption induced by indirect transitions in 3C-SiC: (**a**) Different values of ***D***_0_ for ***D***_***ac***_ = 11 eV; (**b**) Different values of ***D***_***ac***_ for ***D***_0_ = 2 × 10^11^ eV/m.

Figure 2(a) and (b) show the degenerate TPA coefficient spectra induced by the indirect transitions in 3C-SiC polytype, for different values of *D*_0_ and *D*_*ac*_ potential deformations^29^,^30^,^31^ (see the Method section). The numerical calculations run as follows: first we use Eq. (1) to calculate the indirect two photon absorption considering the parameters given in Tables 2 and 3, and then the contribution for photon emission and absorption is added for the case of acoustic and intervalley deformation potential scattering (see Eqs (15) and (16)).

Numerical results have been obtained by assuming |***p***_*mv*_|^2^ = 1.1 × 10^−48^ Kg·J and |***p***_*nm*_|^2^ = 6.0 × 10^−52^ Kg·J, according to the theoretical calculations proposed in ref. 32.

The plot of Fig. 2(a) reveals that, for *D*_*ac*_ = 11 eV, the 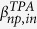 coefficient at 780 nm changes from 0.026 cm/GW to 0.15 cm/GW, with *D*_0_ ranging from 2 × 10^11^ eV/m to 6 × 10^11^ eV/m. Similarly, Fig. 2(b) shows that the TPA coefficient at 780 nm ranges from 0.026 cm/GW to 0.05 cm/GW, if *D*_*ac*_ is changed from 11 to 20 eV, with *D*_0_ = 2 × 10^11^ eV/m. Moreover, our simulations indicate that the indirect TPA process is mainly influenced by the intervalley potential deformation. Indeed, by considering a 10% change in both *D*_0_ and *D*_*ac*_ parameters, we have found a variation in the 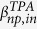 coefficient of about 19.92% and 1.415% for intervalley and acoustic scattering, respectively.

**Figure 3:**
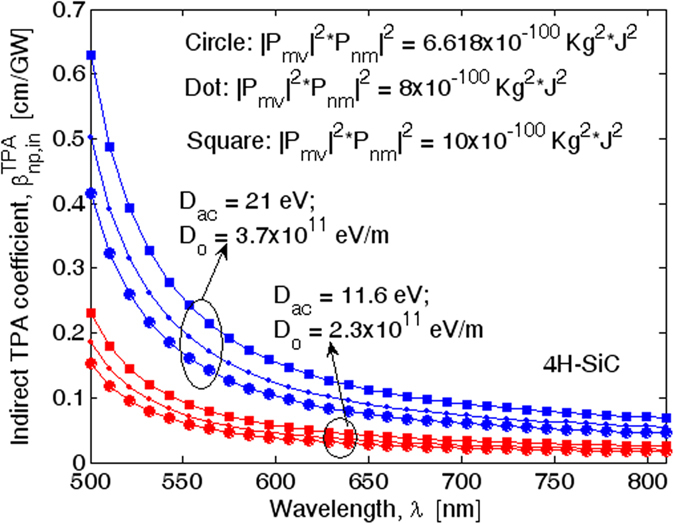
Spectra of degenerate two-photon absorption induced by indirect transitions in 4H-SiC, for different values of the product |*p*_*mv*_|^2^ · |*p*_*nm*_|^2^.

To the best of our knowledge, theoretical or experimental values have not been proposed in the literature for |***p***_*mv*_|^2^ and |***p***_*nm*_|^2^ relevant to the optical transitions 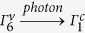 and 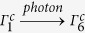 in the 4H-SiC polytype. Thus, in a first approximation, we have assumed for |***p***_*mv*_|^2^ and |***p***_*nm*_|^2^ the same values used for the cubic silicon carbide. In this context, Fig. 3 shows the 4H-SiC indirect TPA coefficient spectra for different values of the product |***p***_*mv*_|^2^ · |***p***_*nm*_|^2^, and assuming two different combinations between the parameters *D*_0_ and *D*_*ac*_ (see Table 3). For the case |***p***_*mv*_|^2^ · |***p***_*nm*_|^2^ = 6.618 × 10^−100^ Kg^2^·J^2^, the plot shows that changing the wavelength in the range [500–810 nm] the 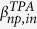 coefficient ranges from 0.416 cm/GW to 0.045cm/GW, and from 0.151 to 0.017 cm/GW for the parameter set *D*_0_ = 3.7 × 10^11^ eV/m, *D*_*ac*_ = 21 eV, and *D*_0_ = 2.3 × 10^11^ eV/m, *D*_*ac*_ = 11.6 eV, respectively. Moreover, the TPA coefficient assumes values of 0.047 cm/GW and 0.0176 cm/GW at 780 nm. These numerical evaluations are consistent with the experimental values of 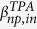 = 0.064 cm/GW at 780 nm^17^. Therefore, we believe that by assuming |***p***_*mv*_|^2^ = 1.1 × 10^−48^ Kg·J and |***p***_*nm*_|^2^ = 6.0 × 10^−52^ Kg·J as for the cubic silicon carbide, consistent information about the indirect TPA process in the 4H-SiC polytype can be achieved.

**Figure 4:**
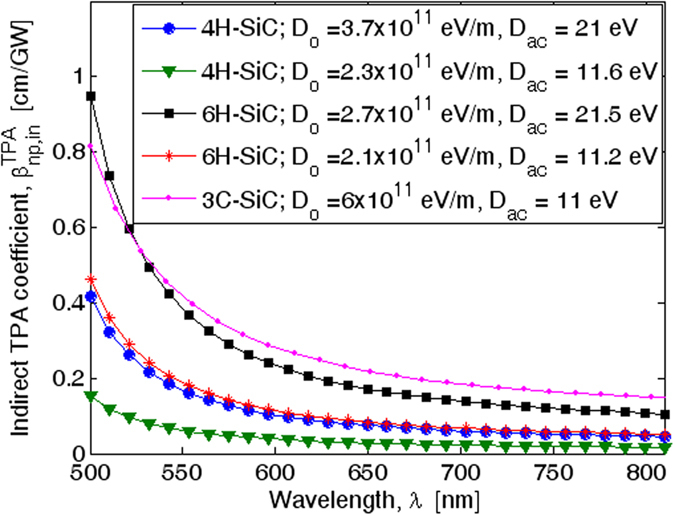
Spectra of degenerate two-photon absorption induced by indirect transitions in 4H-SiC, 6H-SiC, and 3C-SiC.

A comparison of TPA for 4H-SiC, 6H-SiC, and 3C-SiC is shown in Fig. 4, where the indirect TPA spectrum has been simulated assuming the same dipole transition matrix elements (|***p***_*mv*_|^2^ = 1.1 × 10^−48^ Kg·J and |***p***_*nm*_|^2^ = 6.0 × 10^−52^ Kg·J) for all three materials. It is interesting to note that the 6H-SiC curve with *D*_0_ = 2.1 × 10^11^ eV/m and *D*_*ac*_ = 11.2 eV presents 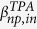 = 0.056 cm/GW at 780 nm, in good agreement with the experimental measurement given in ref. 17, and confirms that the selected parameters can be considered suitable to take into account the electron-phonon interactions in the indirect TPA process. Thus, the plot indicates that the TPA coefficient for 3C-SiC dominates the 6H-SiC one, as a result of the lower value of the indirect energy gap. Therefore, in order to hold the same trend, we guess that 4H-SiC could admit a parameter set such as *D*_0_ = 2.3 × 10^11^ eV/m and *D*_*ac*_ = 11.6 eV to describe the phonon-assisted nonlinear absorption process.

**Figure 5:**
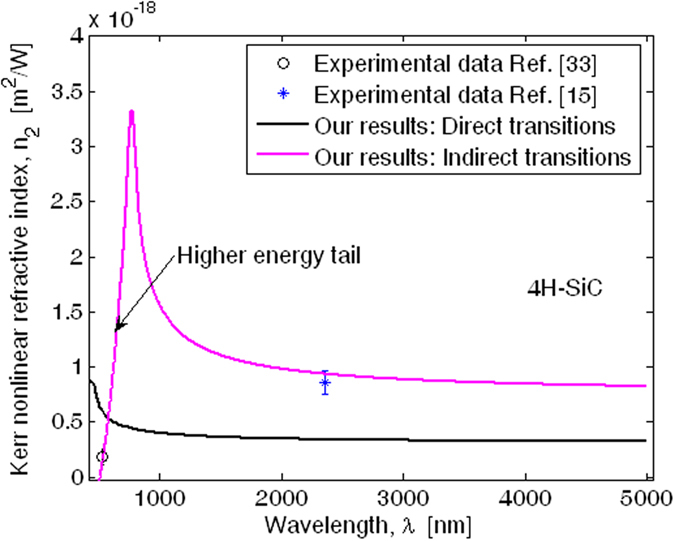
Kerr nonlinear refractive index spectra for 4H-SiC material and TE polarization.

In Fig. 5 the Kerr refractive index (*n*_2_) spectra are sketched for 4H-SiC material and the TE polarization, showing a very good agreement with experimental data^15^,^33^. In the simulations we have assumed a fitting factor *C*_*np*_ = 4.3 × 10^3^ (see Eq. [14]). Moreover, the assumption of nonparabolicity for both conduction and valence bands is demonstrated to be critical in order to match the experimental data. Indeed, our simulations indicate a considerable discrepancy between our theoretical *n*_2_ dispersion and the measurements of refs 15 and 33, if the hypothesis of parabolicity or nonparabolicity only for the conduction band is adopted. Some problems that are linked to the parabolicity hypothesis are: (1) a reduction in the position (*λ*^*peak*^) of the *n*_2_ peak; (2) creation of a smaller tail in the *n*_2_ shape at higher photon energy, and (3) a reduction in the mid-IR asymptotic value. Thus, the matching with experimental data distributed both at “low” and “high” photon energy would thus be definitely compromised. For example, our investigations indicate that *λ*^*peak*^ assumes values of 665.7, 590.9, and 796.4 nm for parabolic, nonparabolic conduction band, and nonparabolic conduction and valence bands, respectively. The *n*_2_ average spectrum induced by the direct transitions is also included in Fig. 5 for comparison. Indeed, generally speaking, 4H-SiC could suffer from the two photon absorption induced by direct transitions, because the direct TPA cut-off wavelength (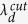 = 482.7 nm) is larger than the transparency wavelength (*λ*^*T*^ = 395.1 nm). The direct transition-induced *n*_2_ has been calculated according to the formula proposed in our previous work^21^ and shows how the typical peak shape^21^ (see also ref. 34) totally disagrees with the experimental data. As a result, we can definitely conclude that the SiC material is dominated by the phonon-assisted two photon absorption.

**Figure 6:**
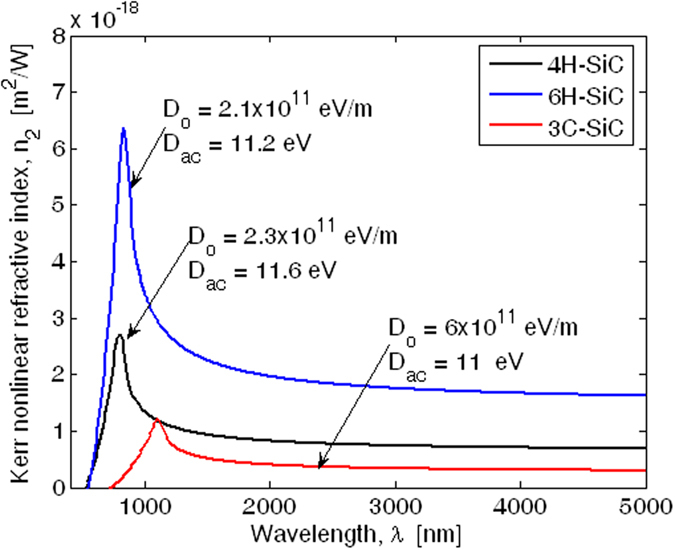
Spectra of Kerr nonlinear refractive index for 4H-SiC, 6H-SiC, and 3C-SiC.

In Fig. 6 the comparison among Kerr refractive indices for 4H-SiC, 6H-SiC, and 3C-SiC is shown. The plot predicts *n*_2_ values in very good agreement with the data proposed in literature, as summarized in Table 3. Over the 500 to 5000 nm range in Fig. 6, *n*_2_ for three carbides is within the range 0.5 to 6.3 × 10^−18^ m^2^/W. It is important to compare this result with that for different materials used in nonlinear photonic applications. An immediate comparison can be made with crystal silicon over the 1500 to 5000 nm wavelength range. We find, by inspecting the red curve-fit-to-data in Fig. 1(a) of ref. 34, that *n*_2_ dispersion for Si is in the range of 0.5 to 5.7 × 10^−18^ m^2^/W. Recently, chalcogenide (ChG) glasses have been proposed in order to fabricate photonic integrated circuits (PICs). They offer unique optical properties for nonlinear optics with a strong Kerr nonlinearity with low two photon absorption and negligible free-carrier effects. These properties have been exploited in a review paper^35^, where recent progress in developing ChG PICs for ultrafast optical processing has been reported. At the same time, hydrogenated amorphous silicon (a-Si:H) has attracted a lot of attention as a platform for nonlinear optics, mainly because it has a larger third order nonlinearity, *n*_2_, compared with other common materials. As outlined in ref. 36, the values for *n*_2_ that have been reported are 4~6 times those of crystalline silicon, 7~13 times those of As_2_S_3_ glass, and 3~5 times those of Ge_11.5_As_24_Se_64.5_. However, a drawback of a-Si:H is that, like crystalline Si, it is reported to suffer from two photon absorption as well as TPA-induced free carrier absorption (FCA) and this ultimately will limit the efficiency of nonlinear devices. In this context, diamond, has recently emerged as a possible platform to combine the advantages of a relatively high nonlinear refractive index, and low nonlinear absorption losses within its large transmission window (from UV to mid-IR)^37^. In Table 4 we summarize the Kerr nonlinear refractive index for the platforms above mentioned.

We believe that the physical model presented here gives practical theoretical predictions and a comprehensive physical overview of the silicon carbide nonlinear nonresonant properties over a wide wavelength range, from visible to mid-IR. Additionally, although only the acoustic phonon deformation potential and the intervalley phonon deformation potential have been considered to describe the complex electron-phonon interactions (see 4H-SiC), the model predictions significantly give consistent results when compared with the number of measurements data available on the third order nonlinearity. Of course, a systematic set of experimental measurements would be useful to better select the values of physical parameters such as 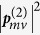, 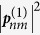, |***Q***_*cn*_|^2^ which have been numerically estimated in this work.

## Conclusions

In this paper, mathematical modeling based on a physical approach has been implemented to investigate the spectrum of two-photon absorption induced by indirect transitions in crystalline silicon carbide having either the cubic or the wurtzite structure. The proposed model has been validated by comparing our predictions with the experimental measurements presented in literature. A group theory analysis has been performed in order to describe the physical features of the phonon-assisted two-photon absorption process. The theoretical investigations have shown that all phonons, except the longitudinal optical phonons, are involved in the process for the 3C-SiC crystal in which the indirect bandgap is induced by the *X* valley. Moreover, phonons with *M*_*2*_ and *M*_*4*_ symmetry located in the both acoustic and optical branches (12 phonons in total) are involved in the indirect TPA process for 4H-SiC and 6H-SiC in which the indirect bandgap is induced by *M* valleys. In order to perform numerical simulations, the complexity of the electron-phonon interactions has been reduced by considering only the acoustic phonon deformation potential and the intervalley phonon deformation potential. However, good agreement with experimental measurement has been achieved, demonstrating the consistency of the physical assumptions adopted. Finally, the Kerr refractive index has been calculated as a function of wavelength for 3C, 4H and 6H. As a result, good agreement between our numerical predictions and experimental measurement has been achieved, demonstrating that the 4H-SiC (6H-SiC) material is dominated by the TPA indirect process, although it could suffer from the direct TPA effect, too. From these results, the silicon carbide can be considered as a very good candidate for nonlinear optical applications since it can guarantee a Kerr effect as large as that of silicon, but without any TPA effect in both the NIR and mid-IR spectral regions.

## Methods

Numerical estimations of |***Q***_*cn*_|^2^ can be obtained by considering only two fundamental scattering mechanisms: acoustic phonon deformation potential scattering (LA phonons), and intervalley phonon deformation potential scattering. In this context, the matrix elements are given by Eqs (15) and (16), respectively:


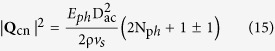



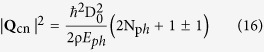


where N_p*h*_ is the photon occupation number given by Eq. (17):


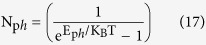


In Eqs (15) and (16), *D*_*ac*_ and *D*_0_ represent the effective acoustic and optical deformation potential, respectively. The coefficients *ρ,* and *v*_*s*_ are the SiC density and the acoustic velocity, respectively. The ± signs correspond to phonon absorption and emission processes. Finally, *E*_*ph*_ represents the acoustic phonon and the intervalley energy in Eqs (15) and (16), respectively. The relevant numerical values used in our simulations are listed in Table 5.

It is worth noting that Eqs (15) and (16) do not describe the exact electron-phonon interaction (especially for the case of 4H-SiC, where complex phonon dispersion occurs), but they still provide useful information about the order of magnitude of |***Q***_*cn*_|^2^.

## Additional Information

**How to cite this article**: Leonardis, F. D. *et al*. Dispersion of nonresonant third-order nonlinearities in Silicon Carbide. *Sci. Rep.* **7**, 40924; doi: 10.1038/srep40924 (2017).

**Publisher's note:** Springer Nature remains neutral with regard to jurisdictional claims in published maps and institutional affiliations.

## Figures and Tables

**Table 1:** The irreducible representation at *M* point.

	*E*	*C*_2_	*σ*_*v*_	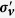
*M*_1_	1	1	1	1
*M*_2_	1	−1	1	−1
*M*_3_	1	1	−1	−1
*M*_4_	1	−1	−1	1

**Table 2:** Parameters of SiC polytypes.

Parameters	3C-SiC	4H-SiC	6H-SiC
*m*_*c*_/*m*_0_	0.37	0.3712	0.71
*m*_*t*_/*m*_0_	0.25	0.42	0.42
*m*_*l*_/*m*_0_	0.67	0.29	0.2
*m*_*v*1_/*m*_0_	1.1	0.99	0.9941
*m*_*v*2_/*m*_0_	0.33	0.9	0.9
*E*_*g,d*_ [eV]	6	5.14	~5.14*
*E*_*g,in*_ [eV]	2.3	3.2	3.0
Δ_*m*_ [eV]	6	5.71	~5.71*
Δ_*n*_ [eV]	6.73	6.2	~6.2*
density [g/cm^3^]	3.21	3.21	3.21
*ε*_*s*_	9.72	9.66	9.66
*d*_*c*_	3	3	6

^*^Since this particular 6H parameters is not known, we have assumed a value close to that of 4H-SiC.

**Table 3:** Estimated Kerr nonlinear refractive index.

Parameters	Our theoretical results *n*_2_ [m^2^/W]	Proposed in literature *n*2 [m^2^/W]
4H-SiC at 532 nm	1.3 × 10^−19^	(1.88 ± 0.7) × 10^−19^ ref. 33
4H-SiC at 2360 nm	9.3 × 10^−19^	(8.6 ± 1.1) × 10^−19^ ref. 15
6H-SiC at 800 nm	6 × 10^−18^	6.14 × 10^−18^ ref. 38
3C-SiC at 1567 nm	4.87 × 10^−19^	4.8 × 10^−19^ ref. 39

**Table 4:** Kerr nonlinear refractive index for different materials.

Materials	Kerr refractive index n_2_ [m^2^/W]
Silicon at 1500–5000 nm	0.5 × 10^−18^–5.7 × 10^−18^ ref. 34
As_2_S_3_ at 1550 nm	3 × 10^−18^ ref. 40; (2.9 ± 0.3) × 10^−18^ ref. 41
(a-Si:H-W) at 1550 nm	2.2 × 10^−17^ ref. 36
Diamond at 1550 nm	(8.2 ± 3.5) × 10^−20^ ref. 37

**Table 5:** Phonon parameters.

3C-SiC polytype	
Parameters	
*v*_*s*_ [m/s]	9.5 × 10^3^
*D*_*ac*_ [eV]	11
*D*_0_ [eV/m]	[2 × 10^11^ ÷ 6 × 10^11^]
Acoustic phonon energy [meV]	a80 (LA phonon)
Intervalley phonon energy [meV]	a99.3 (TO phonon)
4H-SiC polytype	
*v*_*s*_ [m/s]	b1.37 × 10^4^
*D*_*ac*_ [eV]	b11.6–c21
*D*_0_ [eV/m]	b2.3 × 10^11^–c3.7 × 10^11^
Acoustic phonon energy [meV]	a80
Intervalley phonon energy [meV]	b85
6H-SiC polytype	
*v*_*s*_ [m/s]	b1.37 × 10^4^
*D*_*ac*_ [eV]	b11.2–c21.5
*D*_0_ [eV/m]	b2.1 × 10^11^–c2.7 × 10^11^
Acoustic phonon energy [meV]	a80
Intervalley phonon energy [meV]	b85

^a^Ref. 29; ^b^ref. 30; ^c^ref. 31.

## References

[b1] Zorman C. A. . Epitaxial growth of 3C-SiC films on 4 in. diam (100) silicon wafers by atmospheric pressure chemical vapor deposition. J. Appl. Phys. 78, 5136–5138 (1995).

[b2] Song B.-S., Yamada S., Asano T. & Noda S. Demonstration of two-dimensional photonic crystals based on silicon carbide. Opt. Express 19, 11084–11089 (2011). 21716336 10.1364/OE.19.011084

[b3] Yamada S., Song B.-S., Asano T. & Noda S. Silicon carbide-based photonic crystal nanocavities for ultra-broadband operation from infrared to visible wavelengths. Appl. Phys. Lett. 99, 201102 (2011).

[b4] Madar R. Materials science: Silicon carbide in contention. Nature 430, 974–975 (2004). 15329702 10.1038/430974a

[b5] Verma A. P. & Krishna P. Polymorphism and Polytypism in Crystals. (Wiley, New York 1966).

[b6] Choyke W. J. The Physics and Chemistry of Carbides, Nitrides and Borides, NATO ASI Series E. Appl. Sci. 185, 563, Ed. Freer R., Kluwer, Dordrecht (1990).

[b7] Karch K., Wellenhofer G., Pavone P., Röβler U. & Strauch D. Structural and Electronic Properties of SiC polytypes. Paper presented at 22nd Int. Conf. on the Physics of Semiconductors, Vancouver, BC, Canada. Ed.: Lockwood D. J.World Scientific, Singapore (1995).

[b8] Davis R. F. . Critical evaluation of the status of the areas for future research regarding the wide band gap semiconductors diamond, gallium nitride and silicon carbide. Mater. Sci. and Eng B 1, 77–104 (1988).

[b9] Bouclé J. . Local electrooptic effect of the SiC large-sized nanocrystallites incorporated in polymer matrices. Phys. Lett. A 302, 196–202 (2002).

[b10] Kityk I. V., Makowska-Janusik M., Kassiba A. & Plucinski K. J. SiC nanocrystals embedded in oligoetheracrylate photopolymer matrices: new promising nonlinear optical materials. Opt. Mater. 13, 449–453 (2000).

[b11] Borshch A. . Nonlinear refraction in nanocrystalline silicon carbide films. JETP Lett. 88, 386–388 (2008).

[b12] Brodyn M. S. . Nonlinear-optical and structural properties silicon carbide films. J. Exp. Teor. Phys. 114, 205–211 (2012).

[b13] Borshch A. A. . Broadband optical limiting in thin nanostructured silicon carbide films and its nature. Opt. Comm. 364, 88–92 (2016).

[b14] Cheng C.-H. . Can silicon carbide serve as a saturable absorber for passive mode-locked fiber lasers? Scientific Reports 5, 1–15 (2015). 10.1038/srep16463 PMC4642298 26558531

[b15] Cardenas J. . Optical nonlinearities in high-confinement silicon carbide waveguides. Optics Lett. 40, 4138–4141 (2015). 10.1364/OL.40.004138 26368731

[b16] Borshch A. A., Brodin M. S. & Volkov V. I. Self-focusing of ruby-laser radiation in single-crystal silicon carbide. J. Exp. Theor. Phys. 45, 490–492 (1977).

[b17] DesAutels G. L. . Femtosecond laser damage threshold and nonlinear characterization in bulk transparent SiC materials. J. Opt. Soc. Am. B 25, 60–66 (2008).

[b18] Lu X., Lee J. Y., Rogers S. & Lin Q. Optical Kerr nonlinearity in a high-Q silicon carbide microresonator. Opt. Express 22, 30826–30832 (2014). 25607031 10.1364/OE.22.030826

[b19] Tang X., Wongchotigul K. & Spencer M. G. Optical waveguide formed by cubic silicon carbide on sapphire substrates. Appl. Phys. Lett. 58, 917–918 (1991).

[b20] 3C-SiC/Si development. *Anvil Semiconductors* http://www.anvil-semi.co.uk/3c-sicsi-development/ (09/12) (2016).

[b21] De Leonardis F., Troia B., Soref R. & Passaro V. M. N. Dispersion of nonresonant third-order nonlinearities in GeSiSn ternary alloys. Scientific Reports 6, 32622 (2016). 27622979 10.1038/srep32622 PMC5020741

[b22] Dinu M. Dispersion of phonon-assisted nonresonant third-order nonlinearities. IEEE J. Quantum Electron. 39, 1498–1503 (2003).

[b23] Garcia H. & Avanaki K. N. Direct and indirect two-photon absorption in Ge within the effective mass approximation. Appl. Phys. Lett. 100, 2737–2746 (2012).

[b24] Yu P. Y. & Cardona M. Fundamentals of semiconductors: physics and material properties. (Springer-Verlag, Fourth Ed. 2001).

[b25] Lew Yan Voon L. C., Willatzen M., Cardona M. & Christensen N. E. Terms linear in *k* in the band structure of wurtzite-type semiconductors. Phys. Rev. B 57, 10703–10714 (1996). 10.1103/physrevb.53.10703 9982637

[b26] Ivanov I. G. . Phonon replicas at the *M* point in 4*H*-SiC: A theoretical and experimental study. Phys. Rev. B 53, 13633–13647 (1998).

[b27] Shaffer P. T. B. & Naum R. G. Refractive index, dispersion and of Beta silicon carbide. J. Opt. Soc. Am 59, 10498–1498 (1969).

[b28] Shaffer P. T. B. Refractive index, dispersion and birefringence of silicon carbide polytypes. Appl. Opt. 10, 1034–1036 (1971). 20094599 10.1364/AO.10.001034

[b29] Serrano J. . Determination of the phonon dispersion of zinc blende (3C) silicon carbide by inelastic x-ray scattering. Appl. Phys. Lett. 80, 4360–4362 (2002).

[b30] Iwata H. & Itoh K. M. Donor and acceptor concentration dependence of the electron Hall mobility and the Hall scattering factor in n-type 4H– and 6H–SiC. J. Appl. Phys. 89, 6228–6234 (2001).

[b31] Iwata H., Itoh K. M. & Pens G. Theory of the anisotropy of the electron Hall mobility in n-type 4H– and 6H–SiC. J. Appl. Phys. 88, 1956–1961 (2000).

[b32] Willatzen M., Cardona M. & Christensen N. E. Relativistic electronic structure, effective masses, and inversion-asymmetry effects in cubic silicon carbide (3C-SiC). Phy. Rev. B 51, 13150–13161 (1995). 10.1103/physrevb.51.13150 9978113

[b33] Xu C. . Spectral broadening induced by intense ultra-short pulse in 4H–SiC crystals. Chin. Phys. B 25, 064206 (2016).

[b34] Hon N. K., Soref R. A. & Jalali B. The third-order nonlinear optical coefficients of Si, Ge, and Si_1-x_Ge_x_ in the midwave and longwave infrared. J. Appl. Phys. 110, 011301 (2011).

[b35] Eggleton B. J. . Photonic chip based ultrafast optical processing based on high nonlinearity dispersion engineered chalcogenide waveguides. Laser & Photonics Reviews 6, 97–114 (2012).

[b36] Gai X., Choi D. Y. & Luther-Davies B. Negligible nonlinear absorption in hydrogenated amorphous silicon at 1.55 μm for ultra-fast nonlinear signal processing. Opt. Express 22, 9948–9958 (2014). 24787877 10.1364/OE.22.009948

[b37] Hausmann B. J. M., Bulu I., Venkataraman V., Deotare P. & Loncar M. An on-chip diamond optical parametric oscillator’. arXiv:1309.1178v1 [physics.optics] 4 September (2013).

[b38] Jin-Liang D. . Nonlinear Optical Properties and Ultrafast Dynamics of Undoped and Doped Bulk SiC. Chin. Phys. Lett. 27, 124202 (2010).

[b39] Lin Q. Frequency comb generation in SiC microdisk resonators. https://community.apan.org/wg/afosr/m/ultrashort_pulse_laser_matter_interactions_program_review/119124/download (09/12) (2016).

[b40] Zhang Y. . Pump-degenerate phase-sensitive amplification in chalcogenide waveguides. J. Opt. Soc. Am, B 31, 780–787 (2014).

[b41] Madden S. J. . Long, low loss etched As_2_S_3_ chalcogenide waveguides for all-optical signal regeneration. Opt. Express 15, 14414–14421 (2007). 19550720 10.1364/oe.15.014414

